# Lactoferrin-Derived Peptides in Cancer Therapy: Structural Features, Mechanistic Insights and Clinical Translation Prospects

**DOI:** 10.3390/ijms27135702

**Published:** 2026-06-24

**Authors:** Abdulkadir Cidem, Chih-Ching Yen, Ke-Rong Chen, Muhammad Sufian, Gary Ro-Lin Chang, Chuan-Mu Chen

**Affiliations:** 1 Department of Life Sciences, and Ph.D. Program in Translational Medicine, National Chung Hsing University, Taichung 402, Taiwan; cidema.kadir@gmail.com (A.C.); d5210@mail.cmuh.org.tw (C.-C.Y.); coco.kinmen@gmail.com (K.-R.C.); sufianzoologist@gmail.com (M.S.); 2 Department of Molecular Biology and Genetics, Erzurum Technical University, Erzurum 25250, Turkey; 3 Department of Internal Medicine, China Medical University Hospital, and College of Health Care, China Medical University, Taichung 404, Taiwan; 4 Institute of Molecular Biology and Biotechnology (IMBB), The University of Lahore, Lahore 72568, Pakistan; 5 The iEGG and Animal Biotechnology Center, and Rong Hsing Research Center for Translational Medicine, National Chung Hsing University, Taichung 402, Taiwan; 6 Center for General Educational, National Quemoy University, Kinmen 892, Taiwan

**Keywords:** Lactoferrin-derived peptides (LDPs), Lactoferricin (LFcin), anticancer peptides, RRWQWR motif, membrane interaction, apoptosis, immune modulation

## Abstract

Lactoferrin (LF)-derived peptides (LDPs) are short cationic and amphipathic fragments generated primarily from the N-terminal lobe of LF through pepsin-mediated proteolytic processes. The best-characterized LDPs include lactoferricin (LFcin), lactoferrampin (LFampin), and LF1-11. In addition to these native peptides, a growing range of engineered LDPs has been developed by modifying the LFcin-derived RRWQWR motif through the incorporation of non-natural amino acids, cyclization, multimerization, and conjugation with chemotherapeutic agents. LDPs have garnered significant interest as potential anticancer peptides due to their ability to preferentially engage with the surfaces of malignant cells and initiate various tumor-suppressive mechanisms. This review article provides an overview of the principal classes of LDPs and elucidates how structural features influence membrane interaction, selectivity, intracellular targeting, apoptotic pathways, and immune modulation. It also discusses current mechanistic insights and examines the major challenges and opportunities for translating innovative LDPs into clinically useful cancer therapeutics.

## 1. Introduction

Notwithstanding significant progress in cancer research, many current therapies remain limited by dose-limiting toxicities, inadequate tumor selectivity, and the emergence of resistance, thereby fostering sustained interest in alternative therapeutic modalities that can bypass classical resistance pathways. In the realm of cancer research, host-defense peptides have attracted significant attention as innovative therapeutic agents due to their unique ability to selectively target and disrupt cancer cell membranes, thereby not only enhancing therapeutic efficacy but also reducing collateral damage to non-malignant tissues, which signifies a substantial advantage over conventional chemotherapeutics. Moreover, the ability to induce various cell death pathways, such as apoptosis and necrosis, underscores their versatility as therapeutic agents [1,2,3].

Lactoferrin (LF) is a significant reservoir of naturally occurring host-defense peptides that has been extensively studied and utilized. This multifunctional, non-heme iron-binding glycoprotein is abundant in milk and mucosal secretions, and it is recognized for its antimicrobial, immunomodulatory, antiviral, anti-inflammatory, anticancer, and neuroprotective properties [4,5,6]. The proteolytic cleavage of LF during digestion or inflammatory responses leads to the generation of LF-derived peptides (LDPs), which can be divided into three distinct classes: lactoferricin (LFcin) [7], lactoferrampin (LFampin) [8], and LF1–11 [9]. Most LDPs are rich in basic amino acids (specifically Arg and Lys) and aromatic residues (notably Trp and Phe), which facilitates their amphipathic nature and strong interactions with anionic membrane components and proteoglycans; therefore, they were often characterized as antimicrobial peptides (AMPs) during the initial phase of their identification [10,11,12]. These features highlight the potential of LDPs as targeted therapeutic agents in cancer treatment, warranting further investigation into their underlying mechanisms. The earliest work on the anticancer properties of LF was conducted by Bezault et al. in 1994, revealing that oral bovine LF (bLF) administration reduced lung metastasis of B16-BL6 melanoma in mice [13]. Later, Yoo et al. demonstrated the anticancer effects of milk-purified bovine LFcin (LfcinB), emphasizing its inhibitory effects on angiogenesis, tumor growth, and metastasis; additionally, their results exhibited comparable activities between LFcinB (0.5 mg/mouse) and apo-bLF (1 mg/mouse), in contrast to holo-bLF, indicating divergent mechanisms facilitated by LFcinB and the intact bLF protein [14]. The study by Jiang and Lӧnnerdal on colorectal cancer has also demonstrated and compared the anticancer properties of bLF and LFcinB, with LFcinB exhibiting greater tumoricidal efficacy while bLF is linked to signaling pathway modulation and tumor microenvironment adjustments [15]. Overall, the literature supports the perspective that bLF acts as a multifunctional anticancer protein, while LFcinB serves as its more immediately active effector peptide, exhibiting enhanced direct anticancer potency.

Over the past two decades, LDPs have exhibited notable anticancer properties against various types of cancer in preclinical settings. Additionally, advancements in the development of innovative LDPs have revealed the potential to enhance the anticancer potency and specificity of known LDPs. In this review, we outline the structural classifications and sequence–structure–activity interrelationships of LDPs, strategic development methodologies for innovative anticancer LDPs, primary mechanisms of anticancer action, and prospective insights regarding the challenges and clinical translation opportunities in the progression of LDPs.

## 2. Structural Features of LDPs

### 2.1. LFcin

**Figure 1:**
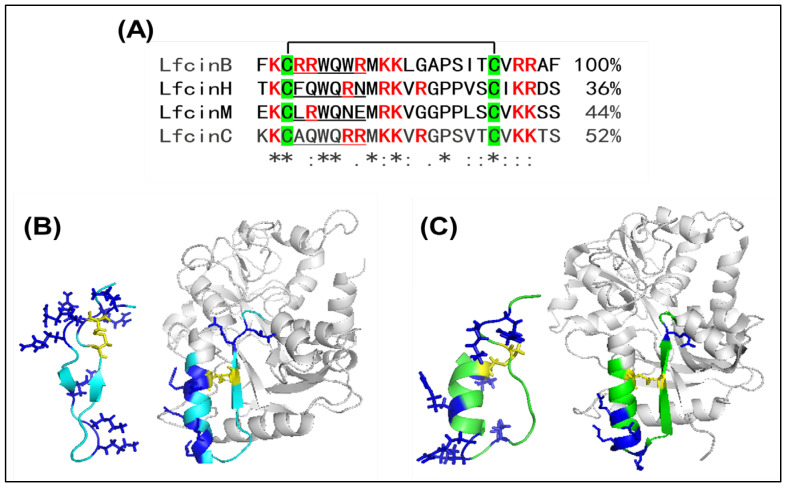
Lactoferricins. (**A**) The alignment of LFcinB, LFcinH, LFcinM, and LfcinC sequences. The residues involved in the disulfide linkage are highlighted in green and connected by lines, while the positively charged residues are marked in red. The motifs related to RRWQWR in LFcinB and the other LFcin variants are underlined. The positions showing amino acid residues that are entirely identical and exhibit a high degree of similarity are denoted by * and :, respectively. (**B**) The NMR-determined LFcinB structure (left) and the 3D structure corresponding to residues 17–41 (right, cyan) in the N1 domain of bLF (gray). (**C**) The NMR-determined LFcinH structure (left) and the 3D structure corresponding to residues 17–41 (green) in the N1 domain of hLF (gray). Structural ribbon representations were generated using PyMOL version 1.7.4, wherein the positively charged residues and disulfide bonds are displayed in blue and yellow stick representations.

LFcin represents the most well-known class of LDPs, released from the N-terminus of LF by pepsin-related proteolysis. Within this class, LFcinB is recognized as the definitive representative, characterized by a 25-residue, highly cationic peptide specified to residues 17–41 in bovine LF (bLF). The identification of LFcinB was first reported in 1992, revealing its superior antibacterial potency relative to intact LF across a broad bacterial spectrum [7,16]. Subsequently, LFcinB has been confirmed to possess inhibitory effects on fungal [17,18], parasitic [19,20], and viral infections [21,22], as well as manifesting protective role in cartilage [23] and anticancer effects in leukemia and breast cancer cells [24,25,26,27,28,29]. These multifaceted effects are attributed to a cationic/aromatic pharmacophore within an 18-residue loop formed by a disulfide bond between Cyc 19 and Cys36, which features a central motif denoted by RRWQWR (LFcin4–9, Figure 1A) [30,31]. The N-terminal region of the LFcinB loop (RRWQWRMKKLG) exhibited similar antimicrobial efficacy compared to full-length LFcinB, but it showed reduced hemolytic activity; moreover, substituting Arg with Lys at the RRWQWR motif partially diminished antimicrobial effectiveness, while the Arg to Glu substitution was found to completely inhibit antimicrobial activities [31]. The RRWQWR core demonstrates structural interaction with sodium dodecyl sulfate (SDS) micelles, showing the entrapment of Trp and Arg residues within the micelles, thereby reinforcing its significance in bacterial membrane interactions [30]. Unlike the full-length LFcinB, the RRWQWR core lacks direct anticancer activity when free; however, upon its introduction into the cytoplasm of T-leukemia or breast cancer cells via fusogenic liposomes, it exhibits strong cytotoxicity with a mechanism different from that of LFcinB [27].

Alongside LFcinB, several LFcin variants from different species have been identified, including LFcinH (humans) [32], LFcinC (camels) [33], and LFcinM (mice) [34]. The alignment shows minimal identity among the sequences of LFcin variants; however, two disulfide bond-forming Cys and certain positively charged Arg and Lys residues are highly conserved (Figure 1A). The solution structure of LFcinB has been resolved by 2D ^1^H NMR (PDB: 1LFC [35]), exhibiting a distorted antiparallel β-sheet configuration, which markedly contrasts with the helical conformation observed in the X-ray crystallography structure of full-length bLF (PDB: 1BLF [36]) (Figure 1B). In contrast to LFcinB, the NMR structure of LFcinH was determined encompassing residues 1–49 (PDB: 1Z6V [37]), revealing a compact β-hairpin/β-sheet topology stabilized by disulfide bonds that is more analogous to the native conformation of human LF (hLF, PDB: 1LFG [38]) (Figure 1C). LFcinH shows antimicrobial and antiviral activity, but it is weaker than LFcinB, despite sequence homology [37,39,40]. The variation is clarified by the spatial arrangement of the β-strand in relation to positive charges through structural modeling, which seemingly influences the activity of divergent LFcin variants [37,39]. LFcinC exhibits a wide antimicrobial range owing to its notable sequence homology with LFcinB [33], yet comparative analyses are deficient. LFcinM is less studied and likely exhibits minimal antibacterial efficacy among LFcin variants [24,34]. In addition to LFcinB and LFcinH, the other LFcin variants have not been rigorously investigated in terms of their anticancer effects.

### 2.2. LFampin

**Figure 2:**
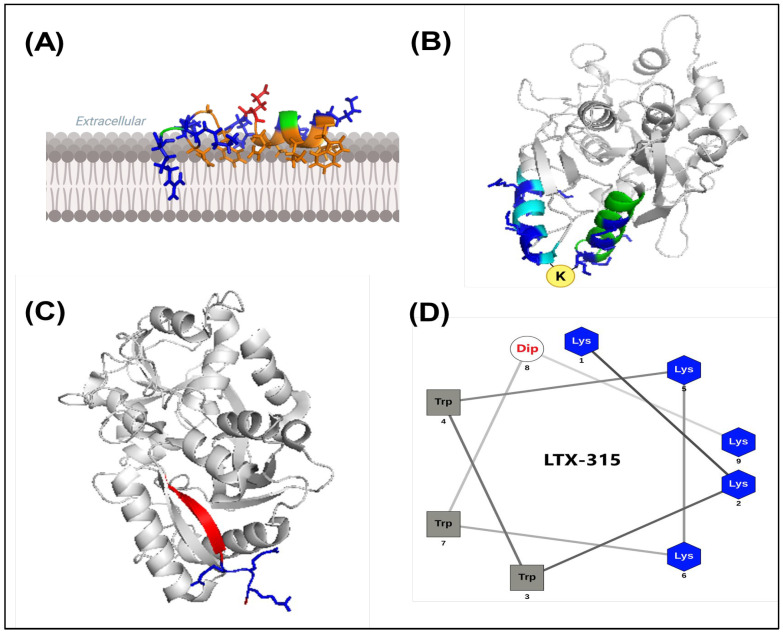
Structural illustration of LFampinB, LFchimera, hLF1-11, and LTX-315. (**A**) Schematic illustration of the interaction between LFampinB and a lipid bilayer. The hydrophobic residues in the N-terminal helix of LFampinB are marked in brown. The amino acid residues exhibiting positive and negative charges are colored in blue and red, respectively, while the remaining residues are depicted in green. (**B**) LFchimera, designed by mimicking the spatial arrangement of LFcin_17–30_ (cyan) and LFampin_265–284_ (green) segments in the N1 domain (gray) of bLF. (**C**) hLF1-11 (red). (**D**) The helical wheel projection of LTX-315 (generated by NetWheels: https://github.com/molx/NetWheels (accessed on 26 January 2026). Structural ribbon representations were generated using PyMOL version 1.7.4, wherein the positively charged residues are displayed in blue stick representations.

The canonical LFampin (LFampinB) is a 17-residue AMP (WKLLSKAQEKFGKNKSR) derived from the N-terminal lobe of bLF (residues 268–284), initially reported in 2004 by van der Kraan et al. [8]. NMR spectroscopy reveals that LFampinB (PDB: 2MD1, 2MD2, 2MD3, 2MD4) features a N-terminal α-helix (W_1_–K_10_) and an unstructured C-terminus (F_11_–R_17_), with the α-helix oriented towards lipid bilayers and its hydrophobic face embedded in the membrane core; the C-terminal positively charged residues are essential for initial attraction to the negatively charged bacterial membrane but do not seem to influence subsequent peptide binding [41,42]. The structural data endorses a two-step antimicrobial model, where the C-terminal positively charged cluster mediates the initial attraction of LFampinB, succeeded by the interaction of the N-terminal helix with the bacterial lipid bilayer interface (Figure 2A) [41,42]. LFampinH, a LFampin variant spanning residues 269–285 (WNLLRQAQEKFGKDKSP) in human LF, has been structurally characterized in SDS micelles by Haney et al. [43], with NMR solution structures demonstrating resemblances to those of LFampinB [41]. These structural findings underscore the association between the membrane orientation of basic and hydrophobic residues and the antimicrobial activity of LFampin-related peptides [41,42,43].

Direct anticancer studies using LFampin itself are limited compared to antibacterial studies, with the most compelling evidence indicating that LFampin contributes to anticancer effects when used in combination with conventional chemotherapeutics or forming a chimeric peptide with LFcin. The first LF-derived chimera (LFchimera) was designed by Bolscher et al. [44], who mimicked the spatial arrangement of LFampin_265–284_ and LFcin_17–30_ segments in the N1 domain of bLF and coupled them to the two amino groups of a Lys residue (Figure 2B), showing better antibacterial activity than individual LFcin and LFampin peptides [44]. A comparable chimeric peptide (LFampin_268–284_ + LFcin_17–30_) along with its antibacterial attributes has likewise been reported [45]. Both LFampin and LFchimera were shown to exhibit considerable anticancer efficacy against HepG2 liver cancer and Jurkat leukemia cells, alongside demonstrate their additive or synergistic effects with cisplatin and etoposide [46]. Recently, LFchimera demonstrated significant anticancer effects against multiple myeloma cells by inducing caspase-independent programmed cell death [47].

### 2.3. LF 1–11

LF1–11 stands for the initial eleven residues in the N-terminus of LF, as reported by Lupetti et al. [48], who were the pioneers in demonstrating the candidacidal properties associated with human LF1–11 (GRRRRSVQWCA). The structure of hLF1–11 is described as a highly cationic N-terminal loop succeeded by a short β-strand in the context of three-dimensional hLF structure (Figure 2C), with the independent NMR structures remain to be resolved. The cationic Arg-rich stretch (R_2_–R_5_) is pivotal to the functionality of hLF1–11, as evidenced by truncation and substitution conducted in this region demonstrating a marked decrease in the affinity for bacterial lipopolysaccharides and antimicrobial efficacy [9,48,49]. The Cys10 residue is also important for hLF–11 activity, especially concerning its immunomodulatory effects on monocytes associating with the inhibition of myeloperoxidase activity [50]. Moreover, hLF1–11 demonstrates immunomodulatory characteristics that promote the differentiation of GM-CSF-stimulated monocyte into macrophages with augmented effector functions [51]. The bovine LF1–11 (APRKNVRWCTI) keeps cationic and hydrophobic properties, yet contains fewer Arg residues and typically has a lower isoelectric point compared to hLF–11 [52,53]. Both hLF–11 and bLF1–11 retain key hydrophobic residues at V6 and W8, signifying a conserved potential for membrane interaction; however, additional Arg residues in hLF–11 likely facilitates enhanced electrostatic binding to negatively charged microbial surfaces and immune targets, potentially leading to superior membrane attachment and immunomodulatory properties than bLF1–11 [52,53].

hLF1–11 itself has not been established as an anticancer peptide in the same regard as LFcinB-derived peptides; however, it can act as a cell-penetrating peptide when conjugated with the core RRWQWR sequence of LFcinB using either a Pro or Gly-Gly linker to increase the cytotoxic impact on Jurkat T-leukemia cells [29]. As there is no reliable evidence for LF1–11 as a direct anticancer peptide, its future promise may be found in cancer-supportive care or alongside other anticancer drugs or peptides to assess whether its immunomodulatory and cell-penetrating properties could enhance anticancer treatment indirectly.

## 3. Milestones in the Development of Innovative LDPs

### 3.1. LFcinB-Based Modification Strategies

Inspired by structure-guided peptide optimization, advanced LDPs exhibiting improved anticancer activity and minimal toxicity to normal cells have been developed. Hilchie et al. [28] developed a novel peptide known as MPLfcinB6 (RRRRRRRGGRRWQWR) by coupling the RRWQWR core of LfcinB to a cell-penetrating hepta-Arg sequence via a Gly-Gly linker and demonstrated that MPLfcinB6 selectively kills human T-leukemia and B-lymphoma cells and exhibits comparable or even greater potency than LFcinB at lower concentrations [28]. The increased positively charged R7 cluster in MPLfcinB6 is shown to be safe; rather, it is believed to facilitate binding to anionic surfaces of cancer cells, allowing the hydrophobic Trp residues to disrupt the cell membrane. Additionally, MPLfcinB6 is nearly half the length of LFcinB, positioning it as a promising therapeutic avenue for treating hematologic malignancies [28]. Utilizing a CLICK chemistry strategy that involves intramolecular cyclization with a triazole bridge, Arias et al. developed LFcinB-CLICK, and demonstrated that this modified version of LFcinB, which replaces the disulfide bridge with a stable triazole linkage, showed similar toxicity to Jurkat cells and exhibited superior cytotoxicity compared to LFcinB against MDA-MB-231 breast cancer cells, suggesting that stable cyclization may enhance activity against more resistant cancer cells [29]. Similar CLICK chemistry approaches have been applied in the synthesis of subsequent innovative LDPs [54,55]. Cárdenas-Martínez et al. [56] reported that dimeric LFcinB-derived peptides with a Met-to-Phe substitution, such as (RRWQWRFKKLG)2-K-Ahx, etc., exhibit fast, selective, persistent, and broad-spectrum cytotoxic effects against a range of cancer cell lines, including colon cancer, prostate cancer, and cervical adenocarcinoma; furthermore, the modifications at Arg residues involving L-ornithine (Orn) or D-Arg residues are shown to enhance anticancer activity while maintaining low toxicity [56]. Ardila-Chantre et al. [57] indicated that the chimeric peptide KKWQWK-Ahx-RLLRRLLR, combining the minimal activity motif of LFcinB, KKWQWK, with the buforin-derived sequence RLLRRLLR, shows considerable promise against cervical cancer cells by inducing rapid, selective, and prolonged apoptosis through an energy-independent internalization mechanism without causing necrosis [57]. Others, such as the tetrameric peptide LFcinB (20–25)_4_, show significant therapeutic potential in the treatment of oral squamous cell carcinoma (OSCC) [58,59] and breast cancer [60,61]. Collectively, LFcinB serves as a fundamental platform for the development of innovative LDPs, and through modifications like residual substitution, optimization, multimerization, and conjugation with cell-penetrating peptides or chemotherapeutic agents, it may show promise in improving anticancer efficacy and selectivity of the developed LDPs.

### 3.2. Innovative LDPs in Clinical Trials

Most of the known LDPs remained in their preclinical trials, with only a very limited number advancing to clinical trials. A clinical study related to hLF1–11 (NCT00509938) has been tested in patients with hematological malignancies to prevent infection during hematopoietic stem cell transplantation, but this pertains to safety and anti-infective applications rather than anticancer assessment [62]. Another instance is LTX-315 (KKWWKKW-Dip-K-NH2), which emerged as a de novo synthetic 9-mer analog of LfcinB with the incorporation of the unusual residue β-diphenylalanine (Dip) and adopting a helix-like structure with five Lys residues on one side and hydrophobic Trp and Dip residues on the other side (Figure 2D) [63]. To the best of our understanding, LTX-315 is the sole LDP that has undergone clinical trials for anticancer assessment, encompassing current Phase I (NCT01986426) and Phase II (NCT03725605) trials. Its anticancer effects were first noted by Camilio et al. [64], demonstrating its effectiveness in controlling melanoma growth by inducing tumor necrosis and robust inflammatory and protective immune responses in mice after intratumoral injection. Later, Haug et al. [63] demonstrated that LTX-315 exhibits significant efficacy against various drug-resistant and drug-sensitive human cancer cell lines, including breast, colon, liver, lung, ovary, and pancreas, among others, with an IC50 below 10 μM and shows limited toxicity to human red blood cells, evidenced by an EC50 over 695 μM; additionally, LTX-315 showed pharmacokinetic properties featuring relatively high plasma protein binding abilities and a human plasma half-life of 160 min, along with a low ability to inhibit CYP450 enzymes. Recently, Fu et al. [65] conducted three rounds of stability-guided optimization, aiming to improve protease resistance and anticancer efficacy of LTX-315 through various modifications involving D-type amino acid substitutions and the creation of hybrid peptides that conjugate camptothecin (CPT), a chemotherapeutic drug with a broad anticancer spectrum but poor solubility. Their initial optimization indicated that D-type peptides like FXY-12 had better proteolytic stability and anticancer effectiveness than L-type LTX-315; additionally, they found that FXY-12 accumulated in mitochondria and significantly reduced mitochondrial membrane potential, demonstrating comparable membranolytic effects to L-type LTX-315. Subsequently. Fu et al. [65] advanced a novel hybrid peptide FXY-30 by conjugating FXY-12 with two CPT moieties and demonstrated that this hybrid peptide exhibited the strongest in vitro and in vivo anticancer activities; furthermore, FXY-30 showed a 700-fold increase in water solubility compared to CPT, along with superior anticancer effectiveness relative to FXY-12, CPT, and LTX-315.

## 4. Preclinical LDP-Associated Anticancer Effects

**Figure 3:**
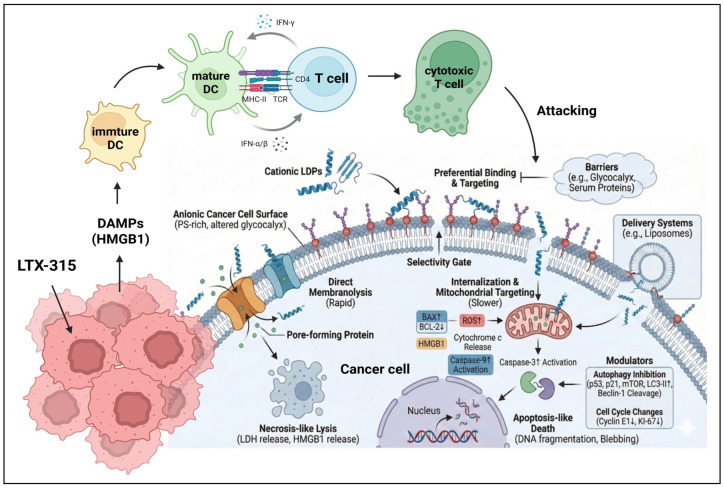
Schematic illustration of the general action mechanisms of LDPs in cancer cells. This illustration was created with BioRender.com (https://BioRender.com/seqoqko, licensed by Kai Cheng Chuang).

Drawing from earlier two-step antimicrobial model and substantial preclinical evidence from diverse cancer cell lines, the structural attributes encompassing both highly cationic and hydrophobic residues endow LDPs with an enhanced affinity with the negatively charged membranes of cancer cells compared to normal cells. The differential interaction yields distinct anticancer effects in both in vitro and in vivo contexts through diverse mechanisms (Figure 3), and Table 1 summarizes the preclinical and clinical outcomes of anticancer research for LDPs, detailing LDP sequences, IC50 values, cancer types, and mechanisms of action.

### 4.1. In Vitro Anticancer Effects

#### 4.1.1. Direct Membrane Disruption

Cancer cells generally show increased levels of anionic phosphatidylserine, enhanced sialylation, and notably glycosaminoglycan side chains (heparan sulfate (HS), chondroitin sulfate (CS), etc.) in their outer cellular membranes, leading to a more negatively charged surface capable of interacting with cationic LDPs [66,67,68]. Earlier work indicated that LFcinB predominantly binds to the surface of cells that express HS, which are present on most cell surfaces [69]; moreover, some cancer cells repair membrane damage less efficiently than normal cells, making them more vulnerable to LDP treatment [70]. Therefore, when LDPs attach to the membrane surfaces of cancer cells, the foremost effect is the alteration of the cellular membrane’s integrity. As confirmed by SEM analysis, cyclic LFcinB (cLFcinB) exhibits a significant capacity to induce direct membrane disruption and lysis in murine Meth A fibrosarcoma cells, a phenomenon absent in treatments involving linear LFcinB [24]. The process of membrane disruption commenced rapidly after cLFcinB incubation, followed by a gradual increase in pore formation indicative of membrane rupture and intracellular content depletion [24]. SEM analysis also revealed significant membrane perforation in MPLfcinB6-treated Jurkat T-leukemia cells, complementary by flow cytometry revealing substantial propidium iodide (PI) and FITC-dextran uptake within 30 min, exemplifying MPLfcinB6’s anticancer action via direct membrane disruption, contrary to caspase-dependent cytotoxicity [28]. The tetrameric peptide LFcinB (20–25)_4_ showed over 90% cytotoxic activity in OSCC cell lines, outperforming linear LFcinB-derived peptides, with treatment causing rapid cell shrinkage and severe membrane damage, leading to cell lysis within an hour and indicating a necrotic process rather than apoptosis [58]. LFchimera treatment was also shown to induce direct membrane disruption and cell lysis in Jurkat and HepG2 cells, unlike the isolated LFcin_17–30_ and LFampin_265–284_ components, which result in apoptosis [46].

Cancer cells may manifest resistance to the oncolytic effects of LDPs through the modifications of surface glycosaminoglycan composition. Earlier work indicated that cells lacking HS were more vulnerable to cLFcinB than those with HS [71]. Additionally, it was observed that exogenous heparin mitigated the cytotoxic impact of cLFcinB, whereas reducing glycosaminoglycan sulfation had the opposite effects, suggesting that longer proteoglycans on the cell surface might keep lytic peptides at a greater distance from the phospholipid bilayer and thereby sequestering their cytotoxic effects [71]. In contrast to the findings with cLFcinB, the cytotoxic effects of three small 9-mer LFcinB-derived lytic peptides (LTX–302, LTX–315, and LTX–318) were shown to be unaffected by cell surface HS, suggesting smaller peptides may be more effective for anticancer treatment [72]. Normal human cells are equipped additional ability to shield themselves from the harmful effects of lytic LDPs by binding these LDPs to the clusters of negative-charged amino acids in the C-terminal lobe of LF binding protein B (LbpB) [73,74].

#### 4.1.2. Induction of Apoptosis

Many studies have highlighted the anticancer effects of LDPs through the induction of apoptosis. In addition to direct membranolytic effects, cLFcinB and linear LFcinB (hereinafter referred to as LFcinB25 for clarity) also induced significant apoptosis in HT-29 colon cancer cells through multiple signaling pathways [15]. However, LFcinB25 has been shown to induce apoptosis in a more diverse array of human cancer cell lines pertaining to leukemia (Jurkat, Raji, K562, CCRF-CEM), breast (MCF-7, T-47D, MD-MB-435), colon (HT-29, Colo-35), and ovarian (Skov3, Caov3) via the mitochondria pathway [25,75]. This process is initiated by the generation of reactive oxygen species (ROS), followed by caspase-2 activation, dissipation of mitochondrial transmembrane potential, release of cytochrome c, and subsequent activation of caspase-9 and caspase-3 [75]. The death receptor-mediated extrinsic pathway was not noted in LFcinB25-induced apoptosis [75]. Ceramide, a membrane sphingolipid acting as a second messenger during apoptosis, was found to be involved in the pro-apoptotic actions of LFcinB25 in T-leukemia cells, where all treatments involving a ceramide analog and inhibitors of ceramidase and glucosylceramide synthase, along with the treatment in the presence of the antiestrogen tamoxifen, enhanced LFcinB25-induced apoptosis, despite LFcinB25 treatment not leading to an increase in cellular ceramide levels [76]. In gastric cancer cells (AGS line), LFcinB25 activated both intrinsic and extrinsic apoptotic pathways, as indicated by the cleavage and activation of caspases-3, -7, -8, and -9, along with poly(ADP-ribose) polymerase (PARP); notably, both apoptosis and autophagy, evidenced by increases in LC3-II and beclin-1 proteins, occurred simultaneously in the early stages of cell death, whereas autophagy was suppressed during the later phases of cell death [77]. Furthermore, LFcinB25 markedly inhibited H460 non-small-cell lung cancer (NSCLC) cell proliferation, suppressed vascular endothelial growth factor (VEGF) expression crucial for angiogenesis and tumor progression, triggered apoptosis via caspase activation and survivin inhibition, and enhanced oxidative stress by increasing ROS while decreasing antioxidant enzyme activity [78].

Shorter LFcinB-derived peptides, such as LFcinB6 (LFcinB_20–25_: RRWQWR), exhibited strong intracellular cytotoxicity in Jurkat and MDA-MB-231 cells when delivered via fusogenic liposomes [27]. The mechanism involved cathepsin B and caspase activation, as inhibitors blocked its cytotoxicity, revealing a distinct apoptotic pathway independent of mitochondrial permeabilization or ROS, contrasting with that of LFcinB25 [27]. LFcinB9 (LFcinB_6–14_) exhibited superior anti-proliferative effects compared to LFcinB25 and significantly induced apoptosis in Skov3 ovarian cancer cells through enhanced ROS production and caspase-3 and caspase-9 activation [79]. LFcinB-P13 (LFcinB_1–13_) showed greater cytotoxic effects than LFcinB25 and induced ROS-mediated caspase-dependent apoptosis in SMMC7721 liver cancer cells [80]. Likewise, TLP18 (LFcinB_1–18_) inhibited cervical cancer cell growth more effectively than LP (identical to LFcinB25), exhibited a stronger affinity for nuclear factor kappaB (NF-κB), and induced apoptosis by reducing NF-κB and increasing NF-κB-interacting long non-coding RNA (lncRNA-NKILA) levels, as evidenced by the attenuation of apoptosis following NKILA silencing, supporting involvement of the lncRNA-NKILA/NF-κB feedback pathway [81].

As shown in Table 1, the in vitro IC_50_ of LDPs generally ranges from 20 to 100 μM after 24 h of treatment, with certain innovative LDPs like LFcinB(20–25)_4_ and LTX-315 exhibiting IC_50_ values under 10 μM.

**Table 1:** Overview of the presented LDPs in conjunction with the relevant in vitro and in vivo research findings.

Peptide	Sequence/ Features	Cancer Cell Lines	IC_50_ (In Vitro)	Effective Dose (In Vivo)	Intervention Outcomes	References
cLFcinB	Cyclic form of LFcinB(17–41): FKCRRWQWRMKKLGAP-SITCVRRAF, cyclization by disulfide bond	Fibrosarcoma (MethA), melanoma (B16F10), and colon carcinoma (C26) [24]. Colon cancer (HT-29) [15]	30 μM for MethA, 70 μM for B16F10, 111 μM for C26, and >500 μM for RBC (24 h treatment) [24].	Fibrosarcoma murine model: intratumoral injection of 500 μg peptides for three consecutive days [24].	Rapid membrane lysis and tumor size regression by nearly 70% [24]. Apoptosis induction via multiple signaling pathways (microarray data) [15].	[15,24]
LFcinB25	Linear form of LFcinB(17–41)	Breast cancer (MDA-MB-435, -231, -468, MCF-7, SKBR3, T47D) [25,26,75]. Leukemia (Jurkat, CCRF-CEM, K562) [75,76] Lymphoma (Raji) [75] Colon (Colo-35, HT-29) [15,75] Ovarian cancer (Skov3, Caov3) [75] Gastric cancer (AGS) [77] Lung cancer (H460) [78] Melanoma (B16-BL6) [14]	32–96 μM for breast cancer cells (24 h treatment): SKBR3>MDA- MB- 231>MDA- MB- 468>MCF7 [25]. ~32 μM (12 h treatment) for leukemia cells [75,76]. 64 μM for AGS (24 h treatment) [77]. ~20 μM (24 h treatment) for H460 cells [78].	MDA-MB-231 xenograft murine model: three doses (4 or 5 mg/day) [26]. H460 xenograft murine model: 75 mg/kg tri-weekly over three weeks (67% inhibitory rate) [78]. Metastasis murine models: subcutaneous administration of a single dose of 0.5 mg (short-term model) or intravenous injection of three doses of 0.5 mg at 3-day intervals (long-term model) [14].	Apoptosis induction. Specifically, through the mitochondrial pathway [75]. C6 ceramide and tamoxifen enhanced LFcinB’s effects in ER-negative breast cancer cells and T-leukemia cells [25,76]. Significant tumor size reduction [26]. Autophagy plays a role in early LFcinB25-induced AGS cell death but is inhibited during late apoptosis [77]. Inhibition of VEGF and Survivin expression [78]. Production of ROS [75,78]. Inhibition of tumor growth, angiogenesis, and metastasis [14]	[14,15,25,26,75,76,77,78]
MPLfcinB6	RRRRRRRGGRRWQWR	Leukemia (Jurkat, CCRF-CEM), lymphoma (Raji, Ramos)	25–50 μM for Jurkat, CEM, and Raji cells; > 50 μM for Ramos cells (24 h treatment); low cytotoxicity to normal T cells	No data (ND)	Membranolytic effects; sequential ROS production and mitochondrial membrane permeabilization, but both are independent of peptide-induced cell death.	[28]
LFcinB-CLICK	FK*RRWQWRMKKLGAP-SIT*VRRAF, cyclization by triazole (*) linkage	Leukemia (Jurkat); breast cancer (MDA-MB-231)	<40 μM for Jurkat, 45% cytotoxicity against MDA-MB-231 at 40 µM (24 h treatment); minimal cytotoxicity to PBMCs and RBCs	ND	Induction of membrane permeabilization in the presence of negatively charged model membrane.	[29]
Dimeric LFcinB(20–30)_2_ peptides	(RRWQWRFKKLG)_2_-K-Ahx (26[F]) and other 26[F]-derived peptides with *L*-Orn an/or *D*-Arg substitutions at various sites	Colon cancer (HT-29 and Caco-2), prostate cancer (DU-145), and cervical cancer (HeLa)	26[F]: 12 μM for HT-29, 18 μM for Caco-2, 17 μM for DU-145, and 7 μM for HeLa (2 h treatment) Peptide #3 ((R-Orn-WQWRFKKLG)_2_-K-Ahx) emerged as a promising candidate with comparable effects.	ND	Apoptosis induction.	[56]
LFcinB/Buforin chimera	KKWQWK-Ahx-RLLRRLLR (CH-1)	Cervical cancer (HeLa, Ca Ski)	16 μM for HeLa, and 14 μM for Ca Ski after 2 h treatment	ND	Apoptosis induction.	[57]
Tetrameric LFcinB(20–25)_4_ peptide	(RRWQWR)_4_-K_2_-(Ahx)_2_-C_2_	1. OSCC (CAL27, SCC15) [58,59] 2. Breast cancer (MCF-7, MDA-MB-231, MDA-MB-468) [60,61]	9 μM for OSCC cells [58], and 6–15 μM for breast cancer cells (24 h treatment) [60,61]	DMBA-induced OSCC hamster model: Chronic treatment (3 times per week) with 15 doses of 30 μg peptides [59].	Apoptosis induction [58,59,60]. Mitochondrial membrane depolarization and intracellular calcium overload [60]. Significant tumor size reduction and increased apoptosis in OSCC tumors [59].	[58,59,60,61]
LTX-315	KKWWKKW-Dip-K-NH_2_	1. Various cancer cell lines from blood, brain, breast, colon, kidney, liver, lung, lymphoma, ovary, pancreas, prostate, and skin [63] 2. Melanoma (B16F1, Fem-X, A375) [64,82,83] 3. Osteosarcoma (U2SO) [84] 4. Transformed rat mesenchymal stem cells (rTMSCs) [85]	1. Mean IC_50_ < 10 μM for various human cancer cells (Panel screening) [63] 2. 12.7–15.3 μM for melanoma cells (4 h treatment) [64]. 3. Time-dependent effects in A375 cells: 30 µM (5 min), 17 µM (60 min) [82]. 4. 7 µM for rTMSCs (2 h treatment) [85]	1. All conducted by intratumoral injection. 2. Melanoma murine model: three doses (1 mg/day) [64,83]. 3. Fibrosarcoma murine model: single dose (300 μg) [84]. 4. Sarcoma rodent model: three doses (1 mg/day) [85] 5. Melanoma murine model: three doses (300 μg/day) [86]	Clinical trials: NCT01986426 (Phase I) [87,88,89], NCT03725605 (Phase II) [90]. Direct membranolytic effects and immunomodulatory effects via the release of ICD-associated DAMPs (such as HMGB1), T-cell infiltration, and proinflammatory cytokine upregulation. Induction of long-term protective immune responses with abscopal effects [85]. CD122-dependent synergistic effects with CTLA4 blockade [86]. Induction of MyD88-dependent tumor-specific DC maturation and migration [83].	[63,64,82,83,84,85,86,87,88,89,90]
LTX-315-derived and hybrid peptides	N-/C-terminal modifications, D-type amino acid substitutions, and hybrid peptides conjugated with CPT	Lymphoma (A20, Daudi, U937), cervical cancer (CaSki, HeLa), ovarian cancer (COC1, ES-2), liver cancer (Hep3B, Huh7), lung cancer (A549/T), breast cancer (MCF-7/ADR), melanoma (B16-F10)	Two peptides were noted with improved IC_50_ values compared to LTX-315: FXY-12 (D-type) and FXY-30 (hybrid). FXY-12: 7.8–182 µM; FXY-30: 3.9–17.9 µM across various cancer cell lines (24 h treatment)	Intratumor injection of FXY-12 and FXY-30 every 4 days for a total of four injections in A20 lymphoma murine model	1. Both FXY-12 and FXY-30 exhibited rapid membranolytic effects and aggregated at mitochondria. 2. FXY-30 induced severe DNA double-strand breaks, leading to cell apoptosis. 3. FXY-12 showed significantly improved proteolytic stability compared to LTX-315, while FXY-30 improved the water solubility of CPT by approximately 700-fold.	[65]
LFampin (265–284)	DLIWKLLSKAQEKFGKNKSR	Liver cancer (HepG2) and T-leukemia (Jurkat).	10 µM for HepG2 (24 h) and Jurkat (4 h treatment).	ND	Treatments induced both apoptosis and necrosis and showed additive or synergistic anticancer effects when combined with cisplatin or etoposide.	[46]
LFchimera (LFampin_265–284_ + LFcin_17–30_)	FKCRRWQWRMKKLG-K-RSKNKGFKEQAKSLLKWILD-NH_2_	Liver cancer (HepG2) and T-leukemia (Jurkat) [46] Multiple myeloma (MM1S, MM1R, and RPMI8226) [47]	10 µM for HepG2 (24 h); 1 µM for Jurkat (6 h treatment) [46]. 3 µM for MM1S and RPMI8226, higher IC_50_ value for MM1R [47]	ND	Treatments induced necrosis and showed additive or synergistic anticancer effects when combined with cisplatin or etoposide [46]. Treatments induced caspase-independent apoptosis in MM cell lines by increasing nuclear translocation of apoptosis-inducing factor and endonuclease G [47].	[46,47]

### 4.2. In Vivo Anticancer Effects

#### 4.2.1. Tumor Regression and Metastasis Suppression

The in vivo anticancer effects of LDPs are typically evaluated in murine models by inducing solid tumors via cancer cell injection, followed by intratumoral LDP administration. Most in vivo studies have shown that LDP intervention effectively inhibits tumor growth, leading to tumor regression or even complete eradication, and in some cases also reduces metastasis depending on the specific LDP, dose, and cancer type. Earlier findings indicated that single subcutaneous administration of 0.5 mg milk-purified LfcinB post-intravenous tumor inoculation significantly diminished liver and spleen lymphoma metastasis and lung melanoma metastasis; furthermore, this intervention markedly suppressed angiogenesis and tumor growth by day 8 post-tumor inoculation; however, the efficacy of LFcinB was not maintained in a prolonged assessment extending to 21 days [14]. Subsequently, intratumoral administration of 500 μg cLFcinB over three days has been shown to result in Meth A tumor size regression by nearly 70% following a 10-day observational period, highlighting the direct lytic effect of cLFcinB on the tumor cells [24]. In MDA-MB-231 breast cancer xenografts, three daily doses of 5 mg LFcinB25 resulted in an approximately 80% decrease in mean tumor volume over a 16-day period [26]. In the context of lung cancer, the triweekly administration of LFcinB25 at a dosage of 75 mg/kg of body weight over three weeks caused a tumor inhibitory rate of approximately 67% in mice bearing H460 tumors [78]. In the OSCC-hamster model, administering LFcinB(20–25)_4_ at a dosage of 30 μg per animal over 15 doses within a span of 5 weeks led to a markedly reduced tumor size compared to the vesicle intervention [59]. Furthermore, by means of a human umbilical vein endothelial cell (HUVEC) model and Matrigel plugs implanted in mice, LFcinB25 demonstrated its inhibitory effects on angiogenesis provoked by basic fibroblast growth factor (bFGF) and VEGF [91]. This mechanism involves competition for heparin-like binding sites on endothelial cells, showcasing an alternative anticancer mechanism of LFcinB by preventing bFGF and VEGF binding to their receptors for pro-angiogenic signaling [91].

#### 4.2.2. Modulation of Tumor Microenvironment and Tumor-Specific Immune Responses

Studies also demonstrate the effects of LDPs on the modulation of tumor microenvironment and tumor-specific immune responses, exemplified by LTX-315, a first-in-class oncolytic peptide-based local immunotherapy. LTX-315 has been shown to induce the hallmarks of immunogenic cell death (ICD) and trigger tumor-specific immune responses after intratumor administration, highlighting the significance of its immune-dependent mechanisms in reshaping the tumor microenvironment. In the settings of melanoma and osteosarcoma, these LTX-315-induced hallmarks of ICD encompass the release of ATP and danger-associated molecular pattern molecules (DAMPs), specifically the high mobility group box-1 protein (HMGB1) from the plasma membrane and mitochondria into the extracellular microenvironment, alongside the exposure of endoplasmic reticulum (ER)-resident calreticulin on the plasma membrane of cancer cells, where it acts as an “eat-me” signal for immune cells to recognize and engulf, as well as type-I interferon responses and notable macrophage and both CD3^+^ and CD8^+^ T cell infiltration, indicating that robust inflammatory responses and enduring protective immunity are triggered [64,82,84,85]. In tumors resistant to intratumoral or systemic cytotoxic T-lymphocyte antigen-4 (CTLA4) checkpoint blockade, subsequent local administration of LTX-315 has been shown to reprogram the tumor microenvironment rapidly, which was characterized by reduced immunosuppressive regulatory T cells (Tregs) and myeloid-derived suppressor cells (MDSCs), concomitantly with increased CD4^+^ helper T cells and CD8^+^ cytotoxic T cells [86]. The intervention of LTX-315 also led to an upregulation of CTLA4 and a downregulation of programmed cell death protein 1 (PD-1) expression levels on T cells, ultimately facilitating tumor regressions with abscopal effects [86]. Furthermore, the synergistic effects between CTLA4 blockade and LTX-315 were demonstrated to be both T cell and the β-chain of the interleukin-2 receptor (CD122)-dependent due to the depletion of CD4^+^ and CD8^+^ T cells, and the neutralizing anti-CD122 monoclonal antibody completely abrogated these effects [86]. Recent investigation has highlighted the critical role of the myeloid differentiation response gene 88 (MyD88) pathway in dendritic cell (DC)-driven immune responses elicited by LTX-315 in a melanoma setting [83]. The results of this research demonstrated that LTX-315 triggered the maturation of tumor-infiltrating DCs and their migration to draining lymph nodes through a multifaceted mechanism involving both direct (LTX-315 forms complexes with DNA fragments released from necrotic tumor cells) and indirect (via tumor-released DAMPs) triggering of multiple Toll-like receptors (TLR7, TLR9) that converge on MyD88 signaling in DCs [83].

## 5. Clinical Translation Prospects of LDPs

### 5.1. Findings from LTX-315

Currently, the main clinical translational insights concerning LDPs in anticancer treatment are derived from LTX-315. The findings of Spicer et al. [87,88,89] indicated that intratumoral LTX-315 injections, both as monotherapy and in combination with immune checkpoint inhibitor (ICI), was generally safe and well-tolerated in patients with advanced solid tumors (NCT01986426). This phase I trial involved dose escalation from 2 to 7 mg per injection, with various treatment schedules tested, including single- and multiple-lesion injections at different frequencies [88]. The predominant grade 3 adverse events include allergic and anaphylactic reactions, but all of which resolved without any lasting complications. In terms of antitumor activity, a significant reduction in tumor volume (≥30%) occurred in 29% of patients, with abscopal effects observed in multiple cases. A total of 44% of evaluable patients displayed stable disease, in contrast to 56% experienced disease progression. The pharmacokinetic data showed plasma concentrations of LTX-315 in most patients declined rapidly to low or undetectable levels within 1 h (T_max_ = 0.02–0.79 h; C_max_ = 6–890 ng/mL); however, the half-life remained undetermined due to insufficient samples. Mechanistically, LTX-315 increased the populations of CD3^+^ and CD8^+^ tumor-infiltrating lymphocytes (TILs) and induced a systemic immune response, with upregulated immune genes involved in tumor regression post-treatment, including those related to effector T cells, T helper type 1 cells, chemokines, and cytokines, substantiating that LTX-315 enriches the tumor microenvironment with adaptive immune components.

Recently, Nielsen et al. presented the outcomes of a phase II clinical trial involving patients with advanced soft tissue sarcoma (NCT03725605) [90]. This trial employed a two-step intervention approach. The initial step involved intratumoral LTX-315 administration at 5 mg, with total doses between 25 mg and 120 mg, and the median interval from the initial injection to surgery for TIL expansion was 21 days. The second step entailed adoptive cell therapy (ACT) and IL-2 treatment, in which ACT was preceded by lymphodepleting chemotherapy and followed by a single intravenous TIL infusion and subsequent subcutaneous administration of IL-2 for a duration of 14 days. Aligning with the findings of Spicer et al. [88], LTX-315 administration was generally well-tolerated, exhibiting manageable grade 1 and 2 adverse events. ACT-related severe adverse events comprised grade 4 neutropenia and thrombocytopenia from lymphodepleting chemotherapy, alongside grade 3 fever associated with TIL infusion and IL-2 therapy. The clinical efficacy was presented with a mean progression-free survival of 22 weeks. The immune responses reported in this phase II trial, including T-cell clonal expansion, tumor microenvironment changes, and TIL expansion, align consistently with the findings from prior preclinical studies and the initial phase I trial.

LTX-315 serves as a prototype oncolytic peptide with a well-characterized mechanism that is likely shared by other LDPs. Nevertheless, clinical validation of other LDPs remains pending, and LTX-315 currently represents the most clinically advanced member of this class to date.

### 5.2. Future Challenges and Research Directions of LDPs

#### 5.2.1. In Vitro to In Vivo Translation

Since the initial identification of LFcinB, the development and research of LDPs have significantly progressed, extending beyond antibacterial properties to encompass anticancer uses. Nonetheless, challenges persist in the implementation of LDPs for anticancer purposes. The greatest challenge in clinical translation is how to convert in vitro results into in vivo efficacy. The main difficulty in this issue stems from the inability of cell culture to accurately replicate the complexities of plasma proteins, extracellular matrix components, immune responses, clearance kinetics, or tissue permeability. Methods such as serum-stability assays, three-dimensional (3D) spheroids, organoids, ex vivo tissue models, and animal models with pharmacokinetic and biodistribution evaluations are increasingly adopted to help identify whether the underlying issue is associated with a reduction in peptide activity, inadequate bioavailability, or insufficient delivery. Among these, 3D spheroids have emerged as critical tools in the high-throughput screening of anticancer agents, offering a more reliable intermediary between in vitro and in vivo testing compared to traditional 2D cultures, although only certain breast, colorectal, pancreatic, melanoma, and lung cancer lines have been refined and standardized for their 3D spheroid cultivation protocols currently [92,93,94,95,96].

#### 5.2.2. Issues of Peptide Stability and Delivery

The second challenge concerns peptide stability, as native peptide sequences are readily degraded by proteases and lose activity in the circulatory system or tumor microenvironment. Earlier milestones have offered some solutions and guidance for forthcoming research. As aforementioned, several innovative LDPs have shown potent cytotoxicity against cancer cells while causing minimal side effects to erythrocytes or other normal cells in preclinical studies. Additionally, their functionality can be fine-tune through straightforward structural changes, including Arg-to-Orn substitution [56], *D*-amino acid incorporation [56,65], multimerization [56,58,59,60], or cyclization [29,54,55], which can enhance both potency and stability. These modified LDPs also provide a range of functionalities. Beyond the direct cytotoxic effects on tumor cells, peptide scaffolds can be adapted for enhanced cellular penetration, targeted delivery, or conjugation with alternative therapeutic agents. Their membrane-interacting approach might further diminish the odds of traditional resistance pathways that restrict the potency of regular small-molecule chemotherapy [97].

Another solution to enhance in vivo efficacy is to encapsulate LDPs within a delivery system that safeguards and concentrates them at the tumor sites. Several methods, including liposomes, fusogenic carriers, nanoparticles, and local depots, can be employed to minimize proteolytic degradation and improve tumor uptake. Earlier, Qi et al. [98] fabricated peptide-loaded nanoparticles (LHPNs) using LFcinB, hyaluronic acid, and poly(acrylamide-co-acrylonitrile)-PEG, with this composition designed to maintain stability under physiological conditions but disintegrate and release LfcinB rapidly in acidic environments (pH 5.5, mimicking lysosomes) and under thermal conditions (43 °C, induced by microwave thermotherapy). LHPN was shown to efficiently delivery and release at tumor sites, exhibiting enhanced cytotoxicity compared to free LFcinB, particularly in conjunction with hyperthermia [98]. This approach presents a promising example for improving the delivery of cationic anticancer peptides and enhancing antitumor immunity.

#### 5.2.3. AI-Assisted Developmental Strategies

Further advancement in LDP development could leverage tumor-specific characteristics rather than relying on a single peptide to demonstrate efficacy universally. This means the need to refine charge, hydrophobicity, and multivalency so that the modified LDP interacts mainly with membranes of cancer cells while leaving normal cells unharmed. In some cases, combining the peptide with chemotherapeutic agents or other bioactive compounds proves to be more efficacious than monotherapy, as synergistic effects can decrease the required dosage and improve the therapeutic window [65]. AI-driven machine learning is becoming a real design tool for peptide discovery, especially for generating short active sequences and balancing activity against toxicity. The optimal computational workflows are likely to integrate sequence-to-activity prediction, membrane-interaction modeling, and toxicity prediction, as these peptides require a balance of efficacy and safety [99]. For de novo generation of LDPs, the recommended workflow can begin with PepINVENT designed for peptide generation beyond natural amino acids [100], followed by PyAMPA for high-throughput screening, mutation, and optimization [101], and then passed to a toxicity predictor such as ToxTeller to remove candidates likely to show undesirable cytotoxicity or off-target risk [102]. In parallel, a membrane- or structure-aware layer is necessary to incorporate amphipathicity, secondary structure propensity, and residue-level features relevant to LFcin-like mechanisms [103]. Optimal LDP candidates can initiate an active learning process to refine models and subsequent designs through continuous retraining with new experimental data.

Future anticancer peptide development will likely focus on three strategies. First, more refined structure–activity optimization is needed, especially using dimeric, cyclic, and short engineered motifs that preserve tumor selectivity while improving stability. Second, peptide design should increasingly be combined with delivery systems such as nanoparticles, tumor-targeted carriers, or local formulations to improve exposure at the tumor site and reduce systemic toxicity. Third, future studies should prioritize mechanism-based validation, including membrane interaction studies, apoptosis and immunogenicity profiling, and animal models that better represent human tumors. From a translational viewpoint, the most realistic opportunities of LDPs are probably in combination therapy and localized delivery, rather than immediate systemic applications. This approach could leverage the benefits of LDPs while addressing significant challenges in clinical development.

## 6. Conclusions

LDPs represent as promising anticancer agents, evolving from their initial identification as antimicrobial peptides. These short, cationic/amphipathic peptides, derived from lactoferrin or engineered based on lactoferricin motifs, exhibit preferential interaction with malignant cell surfaces and trigger various tumor-killing programs. Advanced LDPs, such as LTX-315, have been developed through structure-guided optimization, leading to improved anticancer activity and reduced toxicity to normal cells. The primary mechanisms of LDPs include direct membrane disruption, apoptosis induction, and tumor microenvironment modulation. Continued research into optimizing the sequences of LDPs, chemical interactions, and delivery modalities, based on robust preclinical and clinical studies, will be essential for their successful translation into effective cancer therapeutics.

## Data Availability

No new data were created or analyzed in this study. Data sharing is not applicable to this article.
